# Impact factor: vitamin or poison?

**DOI:** 10.1590/S1516-31802010000400001

**Published:** 2010-07-01

**Affiliations:** I MD, PhD. Full and associate professor, Department of Cardiopneumonology, Faculdade de Medicina da Universidade de São Paulo (FMUSP), São Paulo, Brazil.; II MD. Physician and postgraduate student, Discipline of Thoracic and Cardiovascular Surgery, Faculdade de Medicina da Universidade de São Paulo (FMUSP), São Paulo, Brazil.

Bibliometric indexes are tools that make it possible to measure the production and dissemination of scientific knowledge. Bibliometrics came into being at the beginning of the century because of the need to study scientific production and communication activities both quantitatively and qualitatively.

Among the various indexes that exist, the one that stands out as the most import index is the impact factor, which has been calculated every year since 1972 for the periodicals indexed on the ISI platform (Institute of Scientific Information - Web of Knowledge) and is published in the Journal of Citation Reports (JCR) by Thomson Reuters.^1^

To understand its development, we have to go back to 1963, when Eugene Garfield, the founder of the ISI (today, Thomson Reuters) created the first citation index, the Science Citation Index (SCI). However, Garfield wanted another tool that would, in some manner, be able to assess not only the quantity of articles but also their relevance. The impact factor demonstrates that a given piece of scientific material is of importance, based on the number of times that it has been cited.^2^

The impact factor is calculated as the mean number of citations of articles that were published during the preceding two-year period. For example, the impact factor of a given periodical in 2009 can be calculated as x/y, where x is the number of times that articles published in 2007 and 2008 were cited in indexed periodicals during 2009 and y is the total number of citable items (original articles, review articles, congress abstracts or notes) that were published in 2007 and 2008.

The importance of the impact factor within Brazilian science was suddenly raised when Capes (Coordenação de Aperfeiçoamento de Pessoal de Nível Superior; Coordination Office for Advancement of University-level Personnel) started to use the impact factor as the index of greatest weight for formulating Qualis, a system that was created to assess scientific production and which is used to manage the distribution of financial resources for research funding.^3^ Since then, with the aim of achieving better positioning within Qualis and thus to ensure that funding will be obtained, Brazilian researchers have been increasingly careful in choosing the periodicals in which to publish their papers, such that the impact factor plays a preponderant role in this choice.

This characteristic of authors’ choice has been translated into a new necessity for the editors of Brazilian journals: indexation of their periodicals in the ISI database, as a means of making Brazilian journals competitive and attractive for the national and international scientific communities. This work by Brazilian editors has already borne fruit: in 2008, Brazil only had 31 ISI journals, but in 2009, this number leapt up to 72 ISI journals, an increase of 132%. Another measure of the improvement in the impact of Brazilian periodicals is their position in relation to periodicals in other emerging countries: in 2008, Brazil had eight periodicals among the 100 best classified periodicals within the BRICK grouping (Brazil, Russia, India, China and South Korea), while in 2009, this number went up to eleven.^4^ The São Paulo Medical Journal, for example, has an impact factor of 0.746, according to the current edition of the JCR (2009), thus appearing in sixteenth place among all the Brazilian journals.

Various criticisms regarding the use of the impact factor have been made, and there has been discussion about what the real usefulness of citation measurements is. The validity of the impact factor has even been questioned. This has happened because the index can, in a certain manner, be manipulated through strategies such as self-citation and increasing the allowed number of citations per article.^5^ Another point that has been criticized is that periodicals that publish greater numbers of review articles may obtain higher impact factors than do those that only publish original articles. The number of periodicals within each field of knowledge is also a characteristic that has an impact: a field with fewer journals, such as thoracic surgery, will certainly have journals with impact factors that are lower than those of journals in major fields like cardiology. Therefore, a variety of factors need to be taken into consideration in interpreting the value of the impact factor of a given periodical, and especially with regard to how to use the impact factor in assessments, either of scientists or of institutions. Other less familiar bibliometric tools can be used in an attempt to correct some of the distortions, for example the “Eigenfactor”, which minimizes the modifications caused by self-citation.

Within the Brazilian context, rather than the impact factor itself, its use as the main denominator within Qualis is the matter under discussion. An editorial questioning the criteria of the Qualis system and calling for changes in these criteria was recently published, signed by the editors of a wide range of Brazilian journals.^6^

Nonetheless, there is no argument against the fact that bibliometric indexes are here to stay, since they represent a necessity within modern science. Thus, under the present circumstances, the impact factor constitutes the most widely disseminated tool, thereby justifying its use as the main bibliometric reference, even though it is known to be imperfect.
